# Risk of all-cause mortality by various cigarette smoking indices: A longitudinal study using the Korea National Health Examination Baseline Cohort in South Korea

**DOI:** 10.18332/tid/199670

**Published:** 2025-01-28

**Authors:** Heewon Kang, Eunsil Cheon, Jieun Hwang, Suyoung Jo, Kyoungin Na, Seong Yong Park, Sung-il Cho

**Affiliations:** 1 Institute of Health and Environment, Graduate School of Public Health, Seoul National University, Seoul, Republic of Korea; 2 Department of Public Health Science, Graduate School of Public Health, Seoul National University, Seoul, Republic of Korea; 3 Department of Health Administration, College of Health Science, Dankook University, Cheonan, Republic of Korea; 4 Division of Climate Change and Health Hazard, Korea Disease Control and Prevention Agency, Osong, Republic of Korea; 5 Department of Big Data Service, National Health Insurance Service, Wonju, Republic of Korea

**Keywords:** smoking, intensity, duration, mortality, pack-years

## Abstract

**INTRODUCTION:**

Smoking behaviors can be quantified using various indices. Previous studies have shown that these indices measure and predict health risks differently. Additionally, the choice of measure differs depending on the health outcome of interest. We compared how each smoking index predicted all-cause mortality and assessed the goodness-of-fit of each model.

**METHODS:**

A population-based retrospective cohort, the Korea National Health Examination Baseline Cohort, was used (N=6001607). Data from 2009 were utilized, and the participants were followed until 2021. Cox proportional hazards regression analyses were performed among all participants and ever smokers, respectively, to estimate all-cause mortality. Model fit was assessed by the Akaike Information Criterion.

**RESULTS:**

For men, smoking intensity showed the strongest effect size (hazard ratio HR=1.16; 95% CI: 1.14–1.18), while pack-years provided the best model fit for all-cause mortality. Among women, smoking intensity showed both the strongest effect size (HR=1.49; 95% CI: 1.28–1.74) and the best model fit. Smoking status (never/former/current) also showed comparable effect sizes (men, HR=1.14; 95% CI: 1.13–1.15; women, HR=1.14; 95% CI: 1.11– 1.18) with fair model fit. Analyses of people who ever smoked indicated that a model incorporating smoking status, duration, and intensity best described the mortality data.

**CONCLUSIONS:**

The smoking indices showed varying effect sizes and model fits by sex, making it challenging to recommend a single optimal measure. Smoking intensity may be preferred for capturing cumulative exposure, whereas smoking status is notable for its simplicity, comparable effect size, and model fit. Further research that includes biochemical measurements, additional health outcomes, and longer follow-up periods is needed to refine these findings.

## INTRODUCTION

Estimation of health impacts is a vital aspect of public health. The process of health impact estimation informs public health policy, highlights areas requiring investigation and investment, and assesses the effectiveness of public health policies and programs. Public health policy planning and research priorities should be based on assessments of risk factors and diseases that affect the health of populations. Methodological attempts to better capture health impacts at the population level are always required.

Among the various risk factors for death, tobacco use has, and still does, impose one of the largest disease burdens^1^. The 2019 global estimates found that 7.7 million deaths and 200 million disability-adjusted life years were attributable to tobacco-smoking^1^. Without appropriate interventions, this mortality burden is expected to rise over the coming decades. Thus, the identification of measures that better estimate the health consequences of smoking is a high priority.

Prior epidemiological studies often modeled smoking by categorizing a population as people who have never smoked, formerly smoked, or currently smoke^2^. Smoking prevalence – the proportion of a population that smokes – has also been used in global studies^1^. Other works suggested the use of other smoking measures, such as duration and intensity, when modeling health outcomes^2-5^. In the landmark study of Doll and Peto^6^, various smoking indices were compared in terms of the health outcomes. Such efforts are ongoing, although the results are inconsistent (Supplementary file Tables S1 and S2). Some previous studies, aiming to better capture variation in the dose-response relationship, have independently analyzed individuals with a history of being ever smokers^7,8^, acknowledging that any level of smoking significantly increases health risks compared to being a never smoker.

The optimal measure to use when quantifying the health effects of cigarette smoking remains unclear, as different measures indicate different biological and epidemiological mechanisms. No single measure is perfect, and all measures have unique advantages and disadvantages. Studies comparing the use of different smoking indices for prediction of various health outcomes have yielded different results. For example, one study on US adults aged 45–84 years suggested that smoking intensity (packs/day) provided a better model fit than did smoking status or pack-years when predicting cardiovascular diseases (CVDs)^2^. However, studies focusing on respiratory diseases and lung cancer have generally indicated that smoking duration is a better estimate^4,7,8^ than smoking intensity.

Cumulative smoking exposure has been measured in various ways. Some authors suggested that both pack-years and smoking duration should be used to assess cumulative smoking exposure^5^; others considered that duration alone was a simpler alternative^4^. Various health consequences including lung cancer and CVDs have been modeled employing various smoking indices. To the best of our knowledge, no study has yet used multiple smoking indices to estimate the overall risk of death among the population in the Republic of Korea (hereinafter Korea).

As in other countries, various attempts have been made to estimate mortality or other risks attributable to smoking in Korea^9-13^. One of the first studies to estimate smoking-related deaths reported that the number of deaths caused by smoking in 1985 was 21216 among Korean men and 3122 among Korean women^11^. Subsequent studies reported that the numbers of deaths attributable to smoking were 46208 in 2006^14^ and 58155 in 2012^10^. Most studies reported that the risk of mortality caused by smoking was lower in the Korean population than in Western populations^9-11,13^. It was suggested that this was explained by the heterogeneous smoking patterns of Koreans. Specifically, the age at smoking commencement and the amount of smoking are lower in Korea than Western nations^9^. This implies that smoking behavior should be considered when estimating the health impacts of smoking in Korea. The use of multiple indicators of smoking behavior has provided useful insights in other East Asian countries, including Japan and China, where the smoking risks are also lower than in Western countries^15^. Furthermore, as the data from this study are used to estimate the annual disease burden attributable to smoking in Korea, comparing different smoking indices can provide insights for evaluating this burden.

In this study, we examined how different smoking indices predicted all-cause mortality. Using a large, retrospective, population-based cohort of Korean adults, we compared the estimates and model fits for the overall population and specifically among individuals who ever smoked.

## METHODS

### Data source and study participants

We used retrospective, population-based cohort data, which are a subset of the larger Korea National Health Examination Baseline (KNHEB) Cohort. The KNHEB includes individuals aged ≥19 years and was extracted from the National Health Insurance Database (NHID) of the National Health Insurance Service (NHIS). The NHIS is a universal, mandatory public health insurance system that covers 97% of the Korean population (50 million people)^16^. The NHID includes health screening and death records^17^, thus information on individual cigarette-smoking status and survival. Customized cohort data are available from 2002 onwards. Further details of the database are available elsewhere^16^.

**Figure 1:**
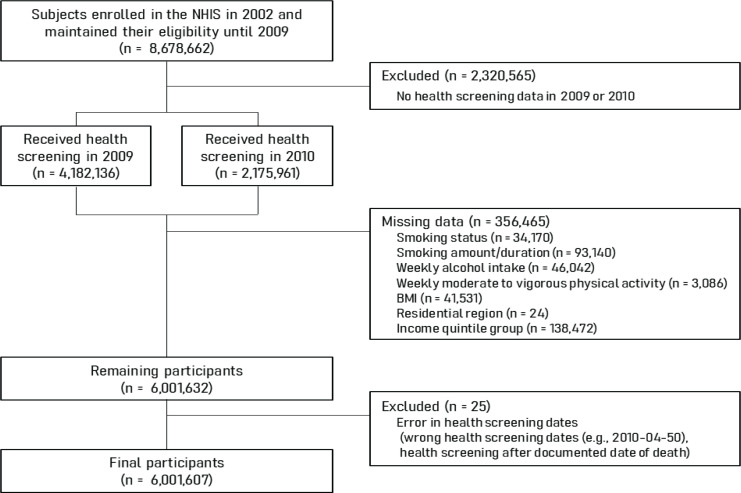
Flowchart of participant selection

The selection of study participants is described in Figure 1. From the NHID, we initially identified those enrolled in the NHIS in 2002 who remained enrolled to 2009 (n=8678662). The decision to follow-up participants from only 2009 reflected the absence of data on smoking duration and intensity before 2009. Biennial health screening is mandatory for all insured Koreans aged ≥20 years, and we used screening data from 2009 (n=4182136) unless health screening data for a participant were available only in 2010 (n=2175961). Participants for whom health screening data were lacking (n=2320565) were excluded; 6358097 individuals remained. A further 356465 were then discarded because of missing data and 25 because of errors in the health screening dates. Finally, 6001607 individuals were included in the analysis.

### Measures

The outcome variable was survival (living or dead; the latter if a date of death had been recorded). The explanatory variable was the smoking behavior at baseline. Six different indices were considered: ever-smoking status, smoking status, current smoking, smoking duration, smoking intensity, and pack-years. All indices have been used in previous studies^2-5,18^ and our data allowed all to be calculated. Measures assessed in previous studies^2,18^ but not here (because the data were lacking) included age at smoking initiation, time since quitting, and a comprehensive smoking index. We used the following definitions for smoking status: 1) ever-smoking, defined as smoking more than 100 cigarettes over a lifetime; 2) smoking status categorized as never, former, and current smoking (former smoking defined as having smoked more than 100 cigarettes but not currently smoking, and current smoking defined as having smoked more than 100 cigarettes and currently smoking daily or occasionally); and 3) current smoking, defined as having smoked more than 100 cigarettes and currently smoking daily or occasionally. Smoking duration and intensity were explored using open-ended questions posed to individuals who currently or formerly smoked. People who currently smoked were asked about their current smoking duration and intensity and those who formerly smoked reported on their duration and intensity before quitting. Pack-years were calculated as the product of smoking intensity (cigarettes/day) and smoking years, divided by 20 (cigarettes/pack). Smoking duration, intensity, and pack-years were categorized based on their respective interquartile ranges.

The covariates were selected based on a previous study on mortality among the Korean population^19^, as well as their availability. These included: age; health insurance type (self-employed, employee-insured, or medical-aid beneficiary); residence (urban, rural); income quintile; weekly alcohol consumption; weekly moderate-to-vigorous physical activity (MVPA) (at least once a week); and body mass index (BMI, kg/m^2^). Age and income quintile were treated as continuous variables and all other parameters as categorical variables.

### Statistical analysis

Descriptive statistics of the study participants at baseline are presented after stratification by both sex and survival status. Kaplan-Meier curves were created by age group (<65 years and ≥65 years) and smoking index for each sex (Supplementary file Figures S1–S6). Cox regression models were then used to investigate the associations between cigarette smoking and death. We assessed the proportional hazards assumption using Schoenfeld residual tests and visual inspections and found that the assumption was not met for all models. Consequently, we applied a time-stratified Cox proportional hazards model, stratifying the follow-up time into quantiles based on event times. Also, all models were tested for multicollinearity, and the variance inflation factor was <5 for all included variables.

For all participants, ever-smoking, smoking status (never/former/current), current smoking (non-current/current), smoking duration, intensity, and pack-years were examined. For ever smokers, five multi-index models (thus using more than one smoking index) were additionally employed: 1) smoking duration and intensity, 2) current smoking and smoking duration, 3) current smoking and smoking intensity, 4) current smoking, smoking duration, and smoking intensity; and 5) current smoking and pack-years. The index combinations were chosen to avoid co-inclusion of indicators that were inherently deducible from another (whole or part) indicator. For example, smoking intensity and duration were not included in any model that considered pack-years, because pack-years are calculated by multiplying intensity and duration.

The participants were followed up from 2009 to 31 December 2021. The goodness-of-fit of each model was assessed using the Akaike Information Criterion (AIC), as in previous studies that employed multiple smoking indices^2,7,18,20^. Lower values of AIC indicate better model fit and an AIC difference >10 is considered substantial^21^. Other measures of model fit, including the Bayesian Information Criterion (BIC) and the concordance index (c-index), were also examined. However, AIC was reported as the primary result, as it is more suitable for model selection than the c-index and does not penalize large sample sizes like BIC. Given the large difference in the male/female smoking prevalence in Korea (4.5% of women and 29.4% of men in 2022), all models were analyzed by sex. All models were adjusted for age, type of health insurance, income quantile, residence, weekly alcohol consumption, weekly MVPA, and BMI.

For models addressing all participants, the effect sizes were compared using the ratio of relative risks (RRR)^22^. For models addressing ever smokers, single-index models were nested within multi-index models, and model fits were compared using log-likelihood ratio tests. All log-likelihood ratio tests indicated a significantly better fit for multi-index models compared to single-index models. Consequently, only the results of multi-index models are reported.

The level of statistical significance for multiple comparisons was adjusted using the Bonferroni method, with a threshold set at 0.0017 (0.05/30). However, Bonferroni corrections were not applied to effect size comparisons or log-likelihood ratio tests, for which the statistical significance level remained at 0.05. All analyses were conducted using R Studio, with the *survival* package applied for Cox models.

## RESULTS

The baseline descriptive characteristics of the study participants (overall and by sex) are shown in Table 1. A total of 6001607 individuals were included, of whom 2383968 (39.7%) were women. The average age was 51.3 years, and men (mean=49.8 years, SD=12.1) were younger than the women (mean=53.5 years, SD=13.1). More than 99% of the participants had health insurance cover. Most (57.5%) lived in urban areas. The average income quintile of men (mean=13.8, SD=5.13) was higher than that of women (mean=12.0, SD=5.74). More men than women drank alcohol weekly (men 67.7% vs women 19.0%) and engaged in weekly MVPA (men 61.0% vs women 44.1%). The proportion with BMI ≥25 kg/m^2^ was 38.0% for men and 28.7% for women.

**Table 1:** Descriptive baseline characteristics of the study participants by sex

*Characteristics*	*Total* *n (%)*	*Men* *n (%)*	*Women* *n (%)*
**Total**	6001607 (100)	3617639 (60.3)	2383968 (39.7)
***Sociodemographic***			
**Age** (years)*	51.3 (50.0) ± 12.7	49.8 (48.0) ± 12.1	53.5 (54.0) ± 13.1
**Health insurance type**			
Self-employed and insured	1181523 (19.7)	574514 (15.9)	607009 (25.5)
Employee-insured	4810640 (80.2)	3039825 (84.0)	1770815 (74.3)
Medical aid beneficiary	9444 (0.2)	3300 (0.1)	6144 (0.3)
**Residence**			
Urban	3450381 (57.5)	2095324 (57.9)	1355057 (56.8)
Rural	740147 (12.3)	395831 (10.9)	344316 (14.4)
Mixed	1811079 (30.2)	1126484 (31.1)	684595 (28.7)
Income quintile*	13.1 (14.0) ± 5.46	13.8 (15.0) ± 5.13	12.0 (13.0) ± 5.74
***Health-related***			
Weekly alcohol consumption	2901005 (48.3)	2447370 (67.7)	453635 (19.0)
Weekly MVPA	3257031 (54.3)	2205941 (61.0)	1051090 (44.1)
**Body mass index** (kg/m^2^)			
<18.5	67416 (2.8)	68666 (1.9)	98750 (4.1)
18.5 to <23	2186279 (36.4)	1148205 (31.7)	1038074 (43.5)
23 to <25	1587824 (26.5)	1024605 (28.3)	563219 (23.6)
≥25	2060088 (34.3)	1376163 (38.0)	683925 (28.7)
**Smoking categories**			
Ever	2511879 (41.9)	2448194 (67.7)	63685 (2.7)
Never	3489728 (58.1)	1169445 (32.3)	2320283 (97.3)
Former	1078149 (18.0)	1055689 (29.2)	22460 (0.9)
Current	1433730 (23.9)	1392505 (38.5)	41225 (1.7)
**Smoking duration** (years)^a^			
≤10	656916 (26.2)	619636 (25.3)	37280 (58.5)
>10 to 20	1021885 (40.7)	1077714 (41.2)	14171 (22.3)
>20 to 25	207892 (8.3)	206406 (8.4)	1486 (2.3)
>25	625186 (24.9)	614438 (25.1)	10748 (16.9)
**Smoking intensity** (cigarettes/day)^a^			
≤10	897973 (35.7)	847290 (34.6)	50683 (79.6)
>10 to 15	389488 (15.5)	385592 (15.8)	3896 (6.1)
>15 to 20	994805 (39.6)	986783 (40.3)	8022 (12.6)
>20	229613 (9.1)	228529 (9.3)	1084 (1.7)
**Pack-years^a^**			
≤7.5	699293 (27.8)	655183 (26.8)	44110 (69.3)
>7.5 to 15	739674 (29.4)	728502 (29.8)	11172 (17.5)
>15 to 22.5	469460 (18.7)	465482 (19.0)	3978 (6.2)
>22.5	603452 (24.0)	599027 (24.5)	4425 (6.9)
**Deaths**	431537 (7.2)	288201 (8.0)	143336 (6.0)
**Follow-up time** (years)*	11.7 (12.1) ± 1.65	11.6 (12.1) ± 1.76	11.7 (12.1) ± 1.47
**Survived** (years)*^b^	12.0 (12.1) ± 0.54	12.1 (12.2) ± 0.53	12.0 (12.1) ± 0.56
**Deceased** (years)*	7.06 (7.42) ± 3.34	6.90 (7.20) ± 3.37	7.38 (7.84) ± 3.28

* Means (median) ± standard deviations of continuous variables.

a Distribution among people who ever smoked (current and former). Smoking duration, intensity, and pack-years were grouped by their interquartile ranges.

b All participants who survived were followed until the end of the follow-up period. Given that mandatory health screenings occur biennially, the follow-up time for surviving participants ranged from 11 to 13 years. All variables showed statistically significant differences by sex (p<0.0001). MVPA: moderateto-vigorous physical activity.

In terms of cigarette use, 67.7% of men and 2.7% of women were ever smokers. Current smoking was recorded for 38.5% of men and 1.7% of women. Smoking duration and intensity among people who ever smoked differed greatly by sex. For men, the highest proportion (41.2%) reported smoking for >10 to 20 years, but most women (58.5%) had smoked for ≤10 years. Also, the smoking intensity of women was lower than that of men. In total, 431537 (7.2%) deaths were observed. During the follow-up period (11.7 years), 8% of men and 6% of women died.

In Table 2, the distribution of deceased and survived groups for each smoking index is presented by sex. Among men, 9.2% of never smokers, 7.7% of former smokers, and 7.1% of current smokers died. For women, the figures were 5.9%, 7.3%, and 11.2%. For both sexes, more individuals with longer smoking durations died compared to those with shorter durations. The death rates were highest for men (8.4%) and women (15.1%) who smoked >20 cigarettes/day compared to those who smoked less. More deaths were identified with higher pack-years for both men (≤7.5 pack-years, 4.1%; > 22.5 pack-years, 14.4%) and women (≤7.5 pack-years, 6.0%; >22.5 pack-years, 27.5%).

**Table 2:** Death or survival by each smoking index, stratified by sex

*Smoking index*	*Men*		*Women*	
	*Deceased*	*Survived*	*Deceased*	*Survived*
	*n (%)*	*n (%)*	*n (%)*	*n (%)*
**Smoking categories**				
Ever	180812 (7.4)	2267382 (92.6)	6258 (9.8)	57427 (90.2)
Never	107389 (9.2)	1062056 (90.8)	137078 (5.9)	2183205 (94.1)
Former	81637 (7.7)	974052 (92.3)	1635 (7.3)	20825 (92.7)
Current	99175 (7.1)	1293330 (92.9)	4623 (11.2)	36602 (88.8)
**Smoking duration** (years)				
≤10	22530 (3.6)	597106 (96.4)	1599 (4.3)	35681 (95.7)
>10 to 20	38775 (3.8)	968939 (96.2)	1188 (8.4)	12983 (91.6)
>20 to 25	8696 (4.2)	197710 (95.8)	132 (8.9)	1354 (91.1)
>25	110811 (18.0)	503627 (82.0)	3339 (31.1)	7409 (68.9)
**Smoking intensity** (cigarettes/day)				
≤10	66458 (7.8)	780832 (92.2)	4737 (9.3)	45946 (90.7)
>10 to 15	17297 (4.5)	368295 (95.5)	283 (7.3)	3613 (92.7)
>15 to 20	77792 (7.9)	908991 (92.1)	1074 (13.4)	6948 (86.6)
>20	19265 (8.4)	209264 (91.6)	164 (15.1)	920 (84.9)
**Pack-years**				
≤7.5	26831 (4.1)	628352 (95.9)	2650 (6.0)	41460 (94.0)
>7.5 to 15	36745 (5.0)	691757 (95.0)	1635 (14.6)	9537 (85.4)
>15 to 22.5	30745 (6.6)	434737 (93.4)	754 (19.0)	3224 (81.0)
>22.5	86491 (14.4)	512536 (85.6)	1219 (27.5)	3206 (72.5)

Among each sex, all variables showed statistically significant differences by survival status (p<0.0001).

**Figure 2:**
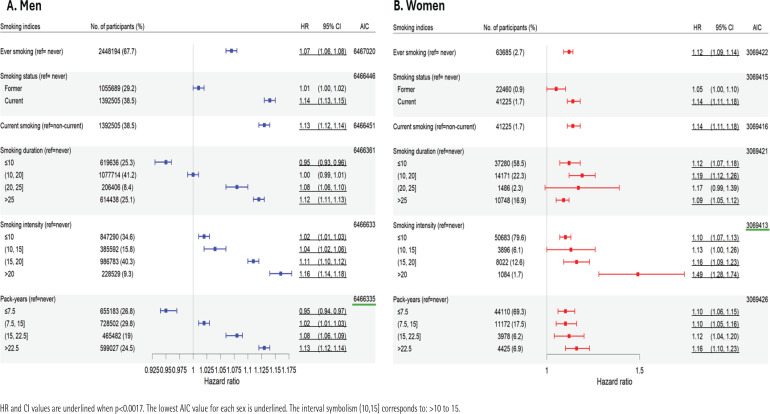
Hazard ratios (HR), 95% confidence intervals (CIs), and the goodness-of-fit values of models assessing the impacts of different smoking measures on all-cause deaths

All smoking indices indicated increased risks for all-cause death (Figure 2 and Supplementary file Table S3). The HRs were somewhat greater for women (range: 1.05–1.49) than men (range: 0.95–1.16). The best-fitting index differed by sex. Pack-years exhibited the best fit for men (AIC=6466335), but smoking intensity the best fit for women (AIC=3069413). The risk of death was highest among individuals who smoked >20 cigarettes/day (men, HR=1.16; 95% CI: 1.14–1.18; women, HR=1.49; 95% CI: 1.28–1.74). Among both men and women, the effect sizes for smoking intensity were significantly greater than those of all other indices (Supplementary file Table S4). Smoking status, smoking intensity, and pack-years exhibited dose-response relationships with all-cause deaths.

Table 3 compares the risk estimates and model fits among individuals who were ever smokers. All models and measures indicated increased risks of all-cause mortality, although some variables lacked statistical significance. For instance, among men, smoking >10 to 15 cigarettes/day did not show statistical significance in Models 1, 3, and 4. However, for both sexes, the highest exposure categories for smoking intensity and pack-years demonstrated significantly positive associations in all models where these indices were included. Furthermore, the model incorporating smoking status, smoking duration, and smoking intensity (Model 4) showed the best fit for both men (AIC=3896424) and women (AIC=91424).

**Table 3:** Hazard ratios and the goodness-of-fits of models assessing the impacts of different smoking measures on all-cause deaths among people who ever smoked

*Variable*	*Model 1*	*Model 2*	*Model 3*	*Model 4*	*Model 5*
	*AHR (95% CI)* *p*	*AHR (95% CI)* *p*	*AHR (95% CI)* *p*	*AHR (95% CI)* *p*	*AHR (95% CI)* *p*
**Men** (N=2448194)					
**Current smoking** (Ref. former)		1.07 (1.06–1.08) **<0.0001**	1.13 (1.11–1.14) **<0.0001**	1.09 (1.08–1.10) 0.0114	1.09 (1.08–1.10) **<0.0001**
**Smoking duration** (years)					
≤10 ®	1	1		1	
>10 to 20	1.04 (1.02–1.05) **<0.0001**	1.04 (1.02–1.06) **<0.0001**		1.02 (1.00–1.04) **<0.0001**	
>20 to 25	1.11 (1.08–1.13) **<0.0001**	1.11 (1.09–1.14) **<0.0001**		1.08 (1.06–1.11) **<0.0001**	
>25	1.16 (1.15–1.18) **<0.0001**	1.15 (1.13–1.17) **<0.0001**		1.11 (1.10–1.13) **<0.0001**	
**Smoking intensity** (cigarettes/day)					
≤10 ®	1		1	1	
>10 to 15	1.00 (0.98–1.02) 0.9705		1.02 (1.00–1.03) 0.0812	1.00 (0.99–1.02) 0.6019	
>15 to 20	1.06 (1.05–1.07) **<0.0001**		1.09 (1.08–1.10) **<0.0001**	1.08 (1.06–1.09) **<0.0001**	
>20	1.11 (1.09–1.12) **<0.0001**		1.16 (1.14–1.18) **<0.0001**	1.13 (1.11–1.15) **<0.0001**	
**Pack-years**					
≤7.5 ®					1
>7.5 to 15					1.06 (1.04–1.07) **<0.0001**
>15 to 22.5					1.10 (1.09–1.12) **<0.0001**
>22.5					1.16 (1.14–1.18) **<0.0001**
**AIC (df)**	3896693 (17)	3896731 (15)	3896666 (15)	3896424 (18)*	3896631 (15)
**BIC**	3896864	3896883	3896818	3896606	3896783
**C-index**	0.831	0.832	0.834	0.833	0.833
**Women** (N=63685)					
**Current smoking** (Ref. former)		1.09 (1.03–1.16) 0.0036	1.13 (1.06–1.19) **<0.0001**	1.11 (1.04–1.18) **0.0010**	1.10 (1.04–1.17) **0.0011**
**Smoking duration** (years)					
≤10 ®	1	1		1	
>10 to 20	1.11 (1.03–1.20) 0.0068	1.10 (1.02–1.19) 0.0137		1.09 (1.01–1.18) 0.0263	
>20 to 25	1.08 (0.90–1.29) 0.3920	1.08 (0.90–1.29) 0.4087		1.06 (0.89–1.27) 0.5121	
>25	1.12 (1.05–1.20) **0.0008**	1.10 (1.02–1.18) 0.0082		1.08 (1.01–1.16) 0.0259	
**Smoking intensity** (cigarettes/day)					
≤10 ®	1		1	1	
>10 to 15	0.98 (0.87–1.11) 0.7384		0.99 (0.88–1.12) 0.8599	0.98 (0.87–1.11) 0.7510	
>15 to 20	1.05 (0.98–1.12) 0.1602		1.07 (1.00–1.14) 0.0537	1.06 (0.99–1.13) 0.1078	
>20	1.35 (1.16–1.58) **0.0002**		1.41 (1.20–1.64) **<0.0001**	1.39 (1.19–1.62) **<0.0001**	
**Pack-years**					
≤7.5 ®					1
>7.5 to 15					1.05 (0.99–1.12) 0.1120
>15 to –22.5					1.08 (0.99–1.17) 0.0804
>22.5					1.14 (1.06–1.22) **0.0002**
**AIC (df)**	91433 (17)	91435 (15)	91425 (15)	91424 (18)*	91430 (15)
**BIC**	91547	91536	91525	91545	91531
**C-index**	0.860	0.860	0.860	0.860	0.860

AHR: adjusted hazard ratio. All models were adjusted for age, the type of health insurance, the income quintile, residence, weekly alcohol consumption, weekly moderate-tovigorous physical activity, and body mass index. Statistical significance is set at p<0.0017, and such values are presented in bold.

* The lowest AIC values of all models for either sex. Each model incorporates different smoking measures. Model 1: smoking duration + smoking intensity. Model 2: current smoking + smoking duration. Model 3: current smoking + smoking intensity. Model 4: current smoking + smoking duration + smoking intensity. Model 5: current smoking + pack-years.

® Reference categories.

## DISCUSSION

We used a large longitudinal cohort of Korean adults to compare the all-cause mortality risk estimates of various smoking indices. Considering both model fit and the strength of association, measures reflecting cumulative exposure, such as smoking intensity, appear to be pragmatic for modeling population-level all-cause mortality risk associated with smoking. For both sexes, smoking intensity (>20 cigarettes/day) showed the strongest association with all-cause mortality. Among men, pack-years provided the best explanation for smoking-related mortality, followed by smoking duration and smoking status. For women, smoking intensity best explained smoking-related mortality, followed by smoking status. As smoking status demonstrated a similar strength of association to smoking intensity and comparable model fit (particularly for women), it may serve as a simple measure for estimating mortality risk when more detailed measures, such as smoking intensity, are unavailable.

Our all-cause death risk estimates by smoking status (HR=1.14 for both currently smoking men and women) were lower than those reported in previous studies of the Korean population. Specifically, they were lower than the estimates from a meta-analysis of four cohorts including the KNHEB (HR=1.73 for men, and HR=1.63 for women). This difference may reflect the shorter follow-up period in our study (maximum 13 years) compared to the data included in the meta-analysis (maximum 28 years)^23^. Furthermore, our estimates were much smaller than those reported in Western populations^24,25^, possibly due to the shorter smoking durations and lower smoking intensities among Koreans. For example, those who smoke >20 cigarettes/day constitute <10% and 2% of Korean men and women, respectively, whereas 58.6% of US men and 46.6% of US women of similar age smoked >20 cigarettes per day^24^.

The risk of all-cause mortality increased across all smoking measures explored in this study. Additionally, measures that reflect the cumulative exposure to cigarette smoking, such as smoking duration, intensity, and pack-years, all showed dose-response relationships with all-cause mortality. These findings contribute to the substantial body of evidence indicating that cigarette smoking compromises health, regardless of how smoking is measured. Despite the large volume of global evidence on smoking-related deaths, deaths from cigarette smoking have been underexplored in Korea. For example, in one meta-analysis assessing the impact of tobacco smoking on mortality in Asia, the estimates for Korea were pooled with those for Singapore and Taiwan because of the small sample size (n=23998)^26^. In contrast, we enrolled over six million participants. However, the limited follow-up period, which was insufficient to fully capture deaths attributable to smoking, highlights the need for continued monitoring of model fit and the strength of associations between smoking indices and health risks.

In contrast to studies on lung cancer^7^ and chronic obstructive pulmonary disease^8^. which reported weaker associations with smoking intensity than smoking duration, we found that smoking intensity exhibited the strongest association with death, in line with previous findings on CVD outcomes^2^. We do not address the contributions of specific diseases to such results; future research on disease-specific mortality using various smoking metrics is required. Also, as cancers exhibit longer latency periods than CVD, our follow-up period may not adequately reflect the impacts of different smoking behaviors. Future studies with longer follow-up periods may show an exacerbated impact of smoking duration with increased cancer risks related to smoking.

Although smoking intensity exhibited stronger associations with all-cause death than smoking duration, intensity is associated with certain limitations that must be addressed to better estimate health risks. First, smoking intensity fluctuates over time, especially with changes in health^27^. Second, smoking intensity is prone to both recall bias^7^ and digit bias (i.e. rounding the smoking intensity up or down)^28^. Even at the same smoking intensity, variations in smoking topography remain challenging when measuring exposure. Factors such as the depth of inhalation, number of puffs taken, and lung smoke retention time are relevant^29^. Although biochemical assessments, including cotinine levels, have often been suggested to directly measure smoke exposure, they do not assess long-term smoking status. Other alternatives, such as wearable sensors^30^, would more accurately measure smoking behaviors in the current era of digital health.

Our analysis of ever smokers provided insights into whether more than one metric should be used to evaluate smoking behavior. Log-likelihood ratio tests showed that models encompassing more than one index demonstrated better model fits for both sexes among ever smokers. Of the multi-index models, the model that included current smoking, smoking duration, and intensity exhibited the best fit. For both sexes, inclusion of smoking status in a model addressing smoking intensity/duration reduced the estimate provided by smoking duration but not smoking intensity. This suggests (again) that smoking intensity is prone to recall bias, especially among those who have quit.

### Limitations

The limitations of this study highlight areas for future research. First, all measures were self-reported at baseline and were therefore subject to recall biases and changes over time. Furthermore, systemic under-reporting of smoking status among Asian women, including Korean women^31^, may have led us to underestimate the risks associated with smoking among women. As a result of these contextual differences, our findings may not be generalizable to other countries. Another factor limiting the generalizability of our findings is the use of AICs as a measure of model fit, which is specific to the data analyzed. However, within the Korean context, AICs provide valuable insights, particularly as the data used in this study are also employed annually to estimate the disease burden from smoking. Secondly, some smoking metrics addressed in previous studies, such as time since quitting, age at smoking commencement, and smoking duration were not examined because the data were lacking. In particular, the age at smoking commencement and the time since quitting would have provided further insights into the cumulative exposure to cigarettes. Residual confounding, including that from the use of other tobacco/nicotine products, may have influenced our results. One report found that those who use more than one tobacco product vary their use intensity^32^. Lastly, the follow-up period (2009–2021) was shorter than the timeframe needed to adequately observe deaths caused by smoking^33^, potentially leading to an underestimation of the mortality risks associated with smoking.

## CONCLUSIONS

In this large longitudinal survey of South Korean adults, we compared different smoking indices in terms of the strengths of association and model fits. Our analyses showed that smoking intensity demonstrated the strongest association and fair model fit, while smoking status also showed comparable strengths of association and model fit, making it a practical and simple measure to prioritize when up-to-date cumulative measures are not available. Identification of a single ‘best’ index remains challenging; as our results indicate that the measures with the best model fit and strongest association vary. Future studies with longer follow-up periods that use additional measures to accurately identify smoking behaviors in Korea are required. All-cause deaths aside, estimations of cause-specific deaths and overall and cause-specific morbidities are warranted.

## Supplementary Material



## Data Availability

All data used in this study can be accessed and analyzed following the approval of National Health Insurance Data Sharing Service of South Korea.
