# Supplementary material for: Risk of all-cause mortality by various cigarette smoking indices: A longitudinal study using the Korea National Health Examination Baseline Cohort in South Korea
Source: Tob Induc Dis. 2025 Jan 28;23:10.18332/tid/199670. doi: 10.18332/tid/199670 (PMC11773640; doi:10.18332/tid/199670)
Supplement: Supplementary file 1 [file TID-23-05-s1.pdf]

SUPPLEMENTARY FILE

Risk of all-cause mortality by various cigarette smoking indices: a longitudinal study using the a longitudinal study using the Korea National Health Examination Baseline Cohort in South Korea

Table S1 Advantages and disadvantages of the various indices used in studies comparing two or more smoking indices

	**Index**							
	**Ever-smoking**	**Smoking status**	**Smoking intensity**	**Smoking duration**	**Pack-year**	**Time since quitting**	**Age at initiation**	**CSI**
Advantages	• Easily quantified and recalled	• Easily quantified and recalled<br>• Comparable to other studies, as this index is most commonly used[1]	• Simple and easily interpreted[2]	• Easily quantified and recalled[3]<br>• Considers cumulative exposure	• The simplest of the composite indices<br>• Considers cumulative exposure in terms of both intensity and duration	• Easily quantified and recalled<br>• Can assess the benefits of quitting early	• Easily quantified and recalled<br>• Implicitly considers cumulative exposure	• Integrates intensity, duration, and time since cessation into a single variable[4]<br>• Avoids multicollinearity between measures[4]
Disadvantages	• Does not distinguish former from current smokers[2,4]	• Does not measure cumulative exposure<br>• Prone to "healthy smoker" biases[4]	• Hard to quantify (e.g., decreases with age and morbidity)[5,6]<br>• Correlates poorly with biochemical assessments[5]	• Does not fully characterize the consumption behaviors of smokers[7]	• Equal weights for amount/ duration[3]<br>• Does not explicitly consider time since quitting[8,9]	• Does not consider consumption behaviors (e.g., duration, intensity)[10]<br>• Collinear with age at the time of smoking initiation and duration[1]	• Does not consider consumption behaviors (e.g., duration, intensity)[10]<br>• Collinear with time since quitting and smoking duration[1]	• Requires additional calculations[11]<br>• Hard to interpret[10,11]

Note: The information presented is a summary of the findings of articles that we initially identified as relevant,[2,7,12,13] and then from backward and forward citation-searching of the Web of Science using these articles. The inclusion criteria were publication in 2000 or later in a peer-reviewed journal, the inclusion of empirical evidence on how at least two smoking indices predicted health outcomes, and explicit comparisons of indices in terms of their advantages, disadvantages, and/or biological mechanisms. The full list of reviewed articles is given in Table S1.
CSI: Cumulative smoking index

Table S2 A summary of previous studies comparing two or more indices when modeling health outcomes associated with cigarette-smoking.

#	First author (published year)	Health outcome examined	Country, setting	Number of participants	Indices included	Authors' conclusions
1	Bhatt 2018[3]	Chronic obstructive pulmonary disease	USA	10,187	• Cigarettes smoked per day (intensity)<br>• Smoking duration<br>• Pack-years	• Smoking duration provided stronger risk estimates than did the smoking intensity or pack-years
2	Flanders 2003[14]	Lung cancer	USA, prospective cohort	116,474	• Cigarettes smoked per day (intensity)<br>• Smoking duration	• Lung cancer death was more strongly associated with smoking duration than cigarettes per day<br>• The association between smoking intensity and mortality decreased with age
3	Hudson 2010[4]	Systemic sclerosis	Canada	606	• Ever-smoking<br>• Smoking status (current/never/former)<br>• Cigarettes smoked per day (intensity)<br>• Smoking duration<br>• Time since quitting<br>• Comprehensive Smoking Index*	• Comprehensive smoking index showed a stronger association and a better model fit compared to other indices<br>• Ever-smoking and smoking status was prone to "healthy smoker" biases
4	Leffondré 2002[1]	Lung cancer	Canada	1,555	• Smoking status (current/never/former)<br>• Cigarettes smoked per day (intensity)<br>• Smoking duration<br>• Cigarette-years<br>• Age at starting<br>• Time since quitting	• Consideration of smoking intensity and duration separately may provide a better fit than cigarette-years<br>• Including cigarette-years when estimating the impact of time since quitting or age at initiation reduced multicollinearity
5	Leffondré 2006[10]	Lung cancer	Canada, case-control study	2,668	• Ever-smoking<br>• Cigarette-years<br>• Time since quitting<br>• Comprehensive Smoking Index*	• No evidence that model fit differed significantly by the index<br>• The Comprehensive Smoking Index may be a useful parsimonious representation of the lifetime smoking history
6	Lubin 2006[15]	Lung cancer	Europe, hospital-based case control study	23,011	• Cigarettes smoked per day (intensity)<br>• Smoking duration<br>• Pack-years	• Intensity showed homogeneous patterns by different histologic types of lung cancer, but showed difference by total exposure and smoking duration

#	First author (published year)	Health outcome examined	Country, setting	Number of participants	Indices included	Authors' conclusions
7	Lubin 2016[16]	CVD	USA, clinic-based prospective cohort study	14,127	• Cigarettes smoked per day (intensity)<br>• Smoking duration<br>• Pack-years	• Pack-years was the primary determinant of smoking-related CVD risks<br>• For those with equal pack-years, smoking fewer cigarettes for longer was more harmful than smoking more cigarettes for shorter durations
8	Lubin 2017[7]	Coronary Heart Disease	USA, prospective cohort study of licensed pesticide applicators and their spouses; Finland, trials of male smokers	89,656; 29,133	• Ever-smoking<br>• Cigarettes smoked per day (intensity)<br>• Pack-years	• Pack-years was associated with coronary heart disease risks<br>• Pack-years and smoking intensities (evaluated together) were inversely associated with intensity; smoking fewer cigarettes for longer was more harmful that smoking more cigarettes for a shorter time
9	Nance 2017[2]	CVD incidence and deaths	USA, multi-ethnic cohort	6,814	• Ever-smoking<br>• Smoking status (current/never/former)<br>• Cigarettes smoked per day (intensity)<br>• Smoking duration<br>• Pack-years<br>• Age at starting smoking<br>• Time since quitting<br>• Comprehensive Smoking Index*	• Current smoking and cigarettes smoked per day exhibited the strongest associations with incidence<br>• Cigarettes smoked per day exhibited a better model fit than current smoking in terms of incidence<br>• The Comprehensive Smoking Index afforded the best fit for CVD deaths
10	Ogawa 2019[12]	Oncogenic mutations in non-small cell lung cancer	Japan, medical institutions	876	• Smoking duration<br>• Pack-years	• Smoking duration and pack-year indices were not significantly different<br>• Smoking duration alone must be considered as a simpler alternative

#	First author (published year)	Health outcome examined	Country, setting	Number of participants	Indices included	Authors' conclusions
11	Remen 2018[6]	Lung cancer	Canada, population based case-control	2,705	• Ever-smoking<br>• Smoking status (current/never/former)<br>• Cigarettes smoked per day (intensity)<br>• Smoking duration<br>• Pack-years<br>• Age at starting<br>• Time since quitting<br>• Comprehensive Smoking Index*	• Smoking duration and the Comprehensive Smoking Index exhibited the strongest associations and the best model fits
12	Young 2019[17]	Peripheral artery disease	North Carolina USA, hospital-based	693	• Packs smoked per day (intensity)<br>• Smoking duration<br>• Pack-years<br>• Time since quitting	• Smoking intensity best identified negative outcomes after open revascularization of patients with peripheral artery disease

* The Comprehensive Smoking Index was originally developed by Hoffman et al.,[18,19] and incorporates smoking intensity, duration, and time since quitting. Studies have used different terms (e.g., Compound Smoking index,[2] Cumulative smoking index[6]) to indicate this measure. However, the formulae used to obtain the estimates were similar across all studies, with slight modifications (log transformations and addition of a lag parameter). These are all termed the "Comprehensive Smoking Index" in this Table.

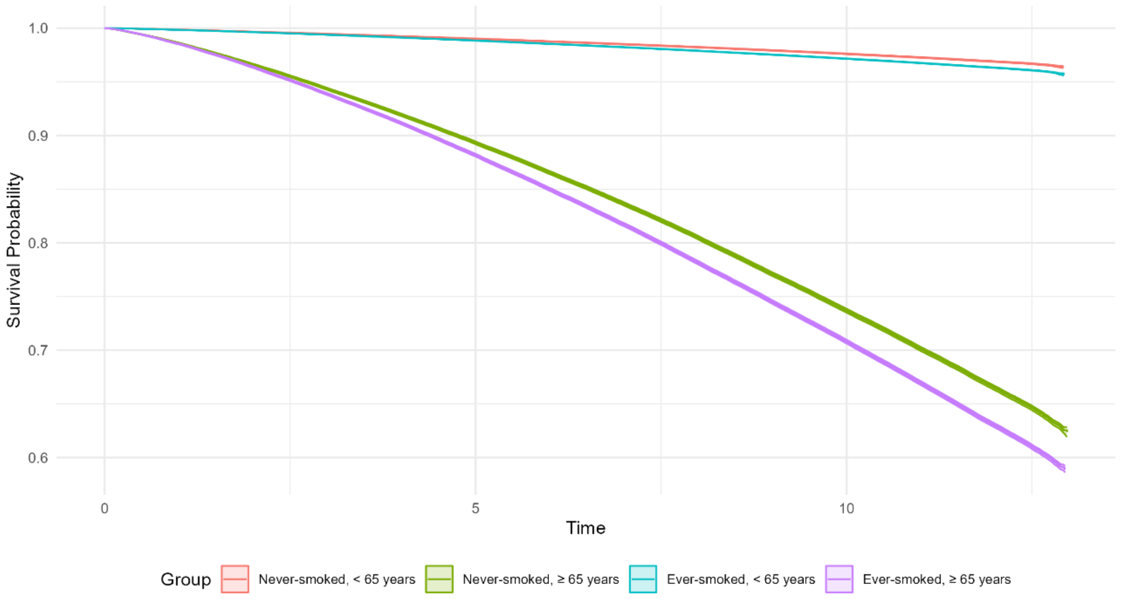

Figure S1.1 Kaplan-Meier curve by age and ever smoking status among men

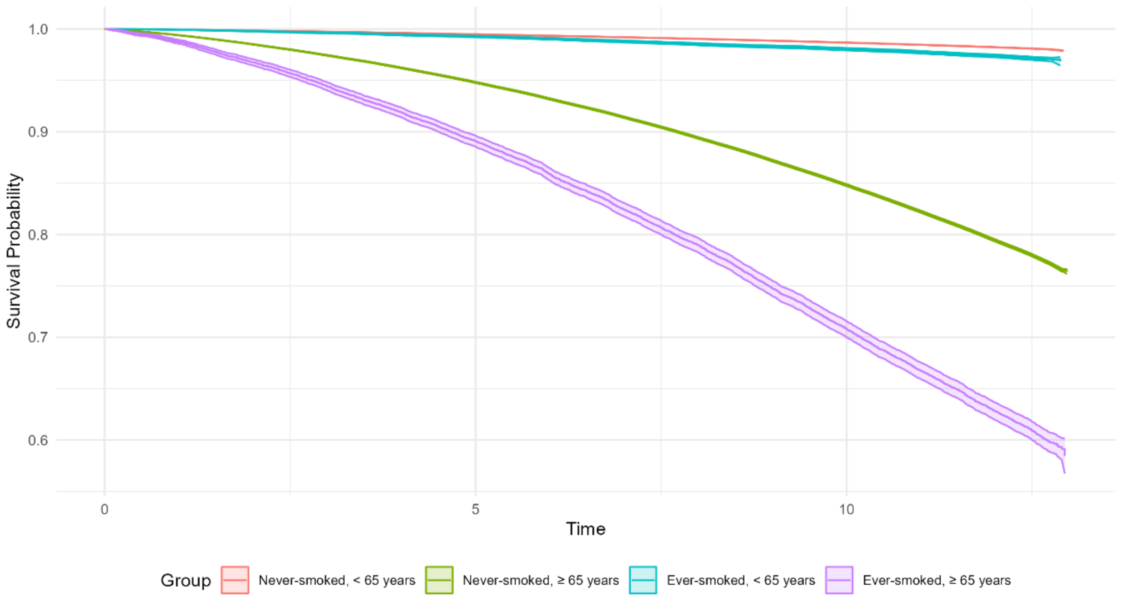

Figure S2.2 Kaplan-Meier curve by age and ever smoking status among women

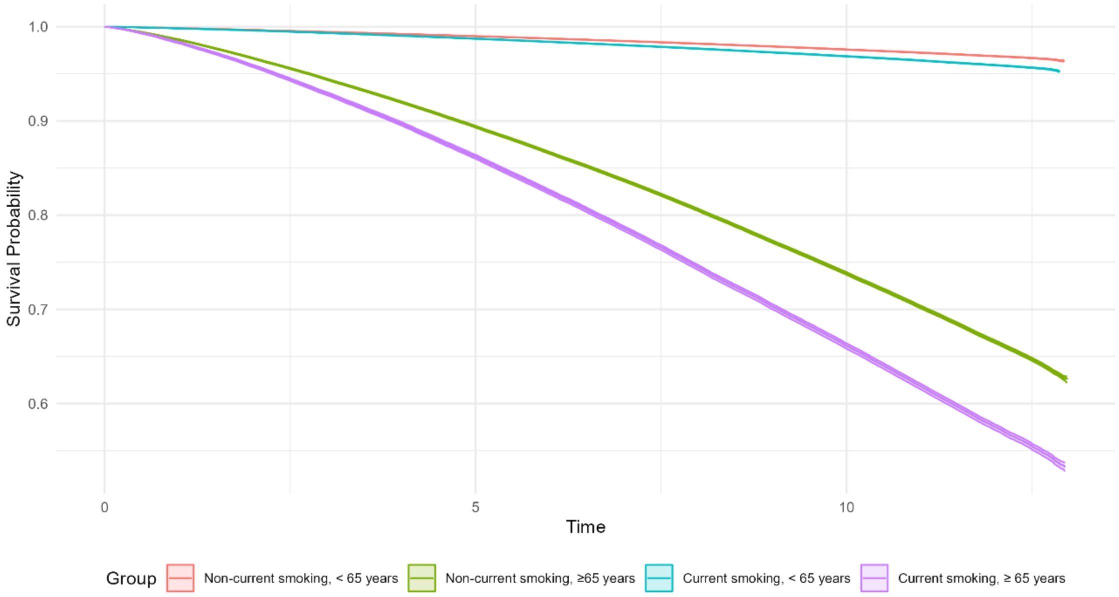

Figure S2.1 Kaplan-Meier curve by age and current smoking status among men

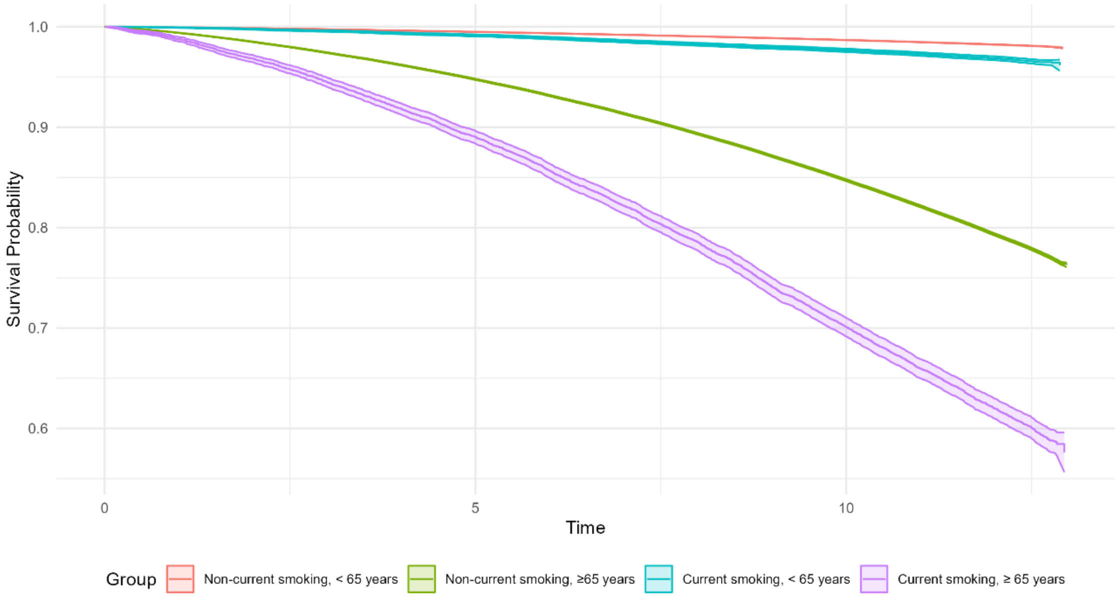

Figure S2.2 Kaplan-Meier curve by age and current smoking status among women

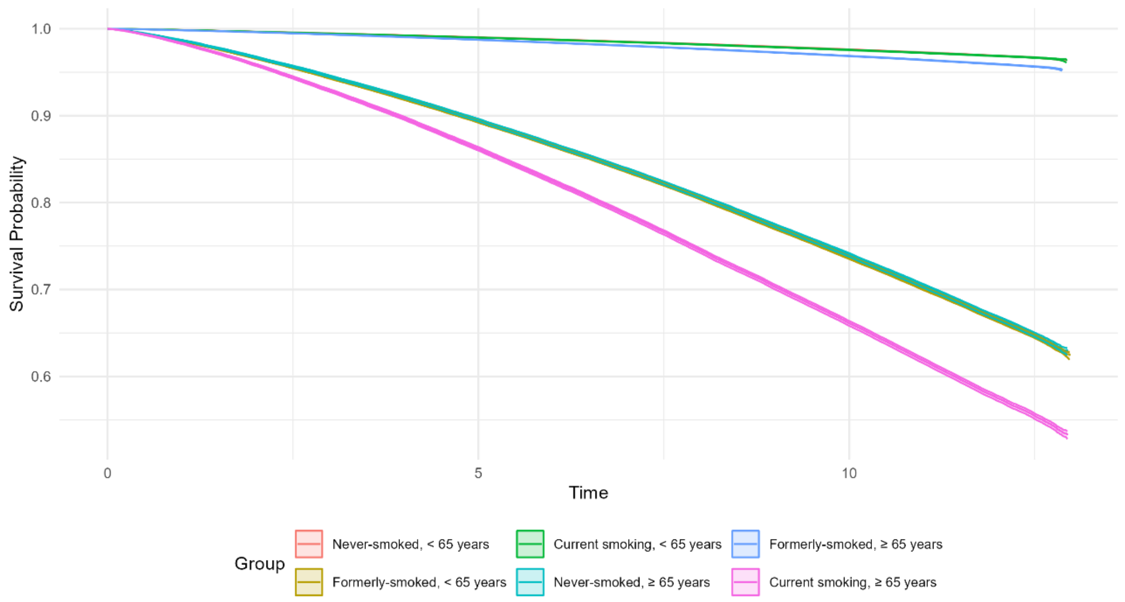

Figure S3.1 Kaplan-Meier curve by age and smoking status (never/former/current) among men

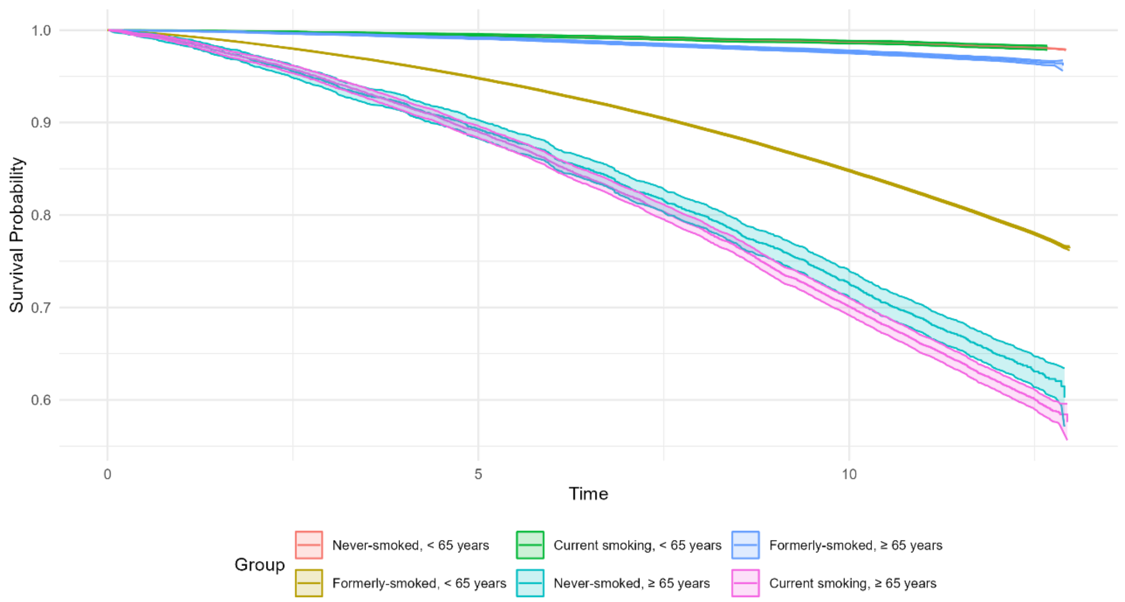

Figure S3.2 Kaplan-Meier curve by age and smoking status (never/former/current) among women

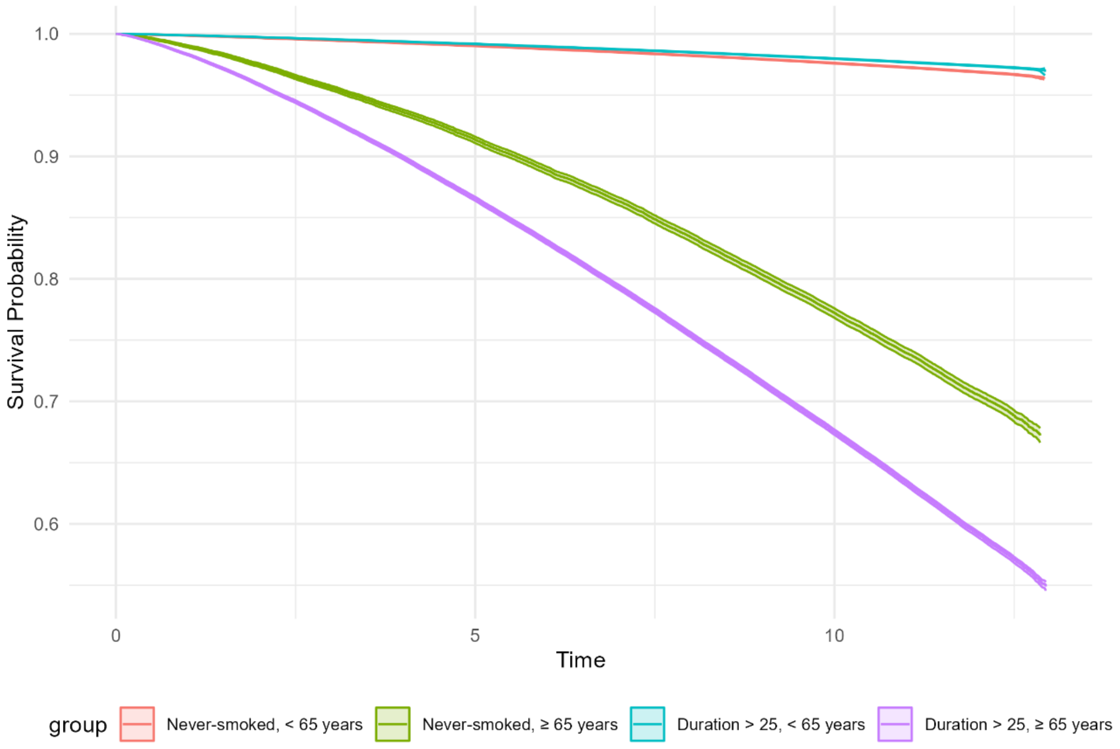

Figure S4.1 Kaplan-Meier curve by age and smoking duration among men

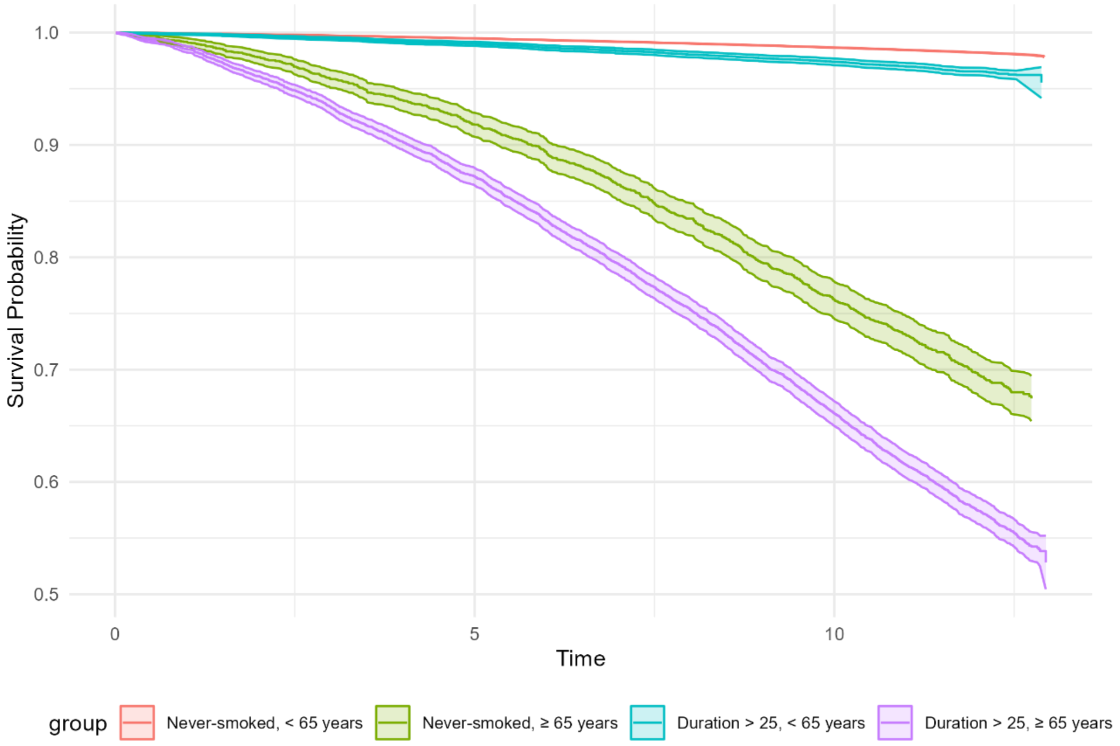

Figure S4.2 Kaplan-Meier curve by age and smoking duration among women

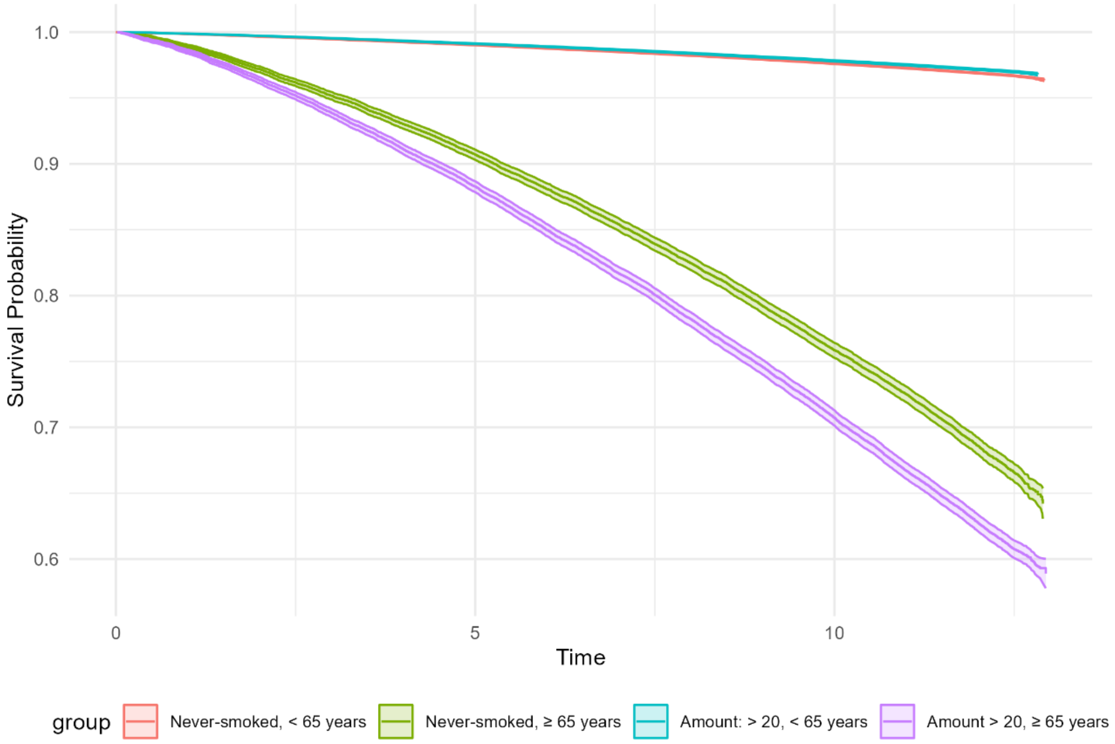

Figure S5.1 Kaplan-Meier curve by age and smoking intensity among men

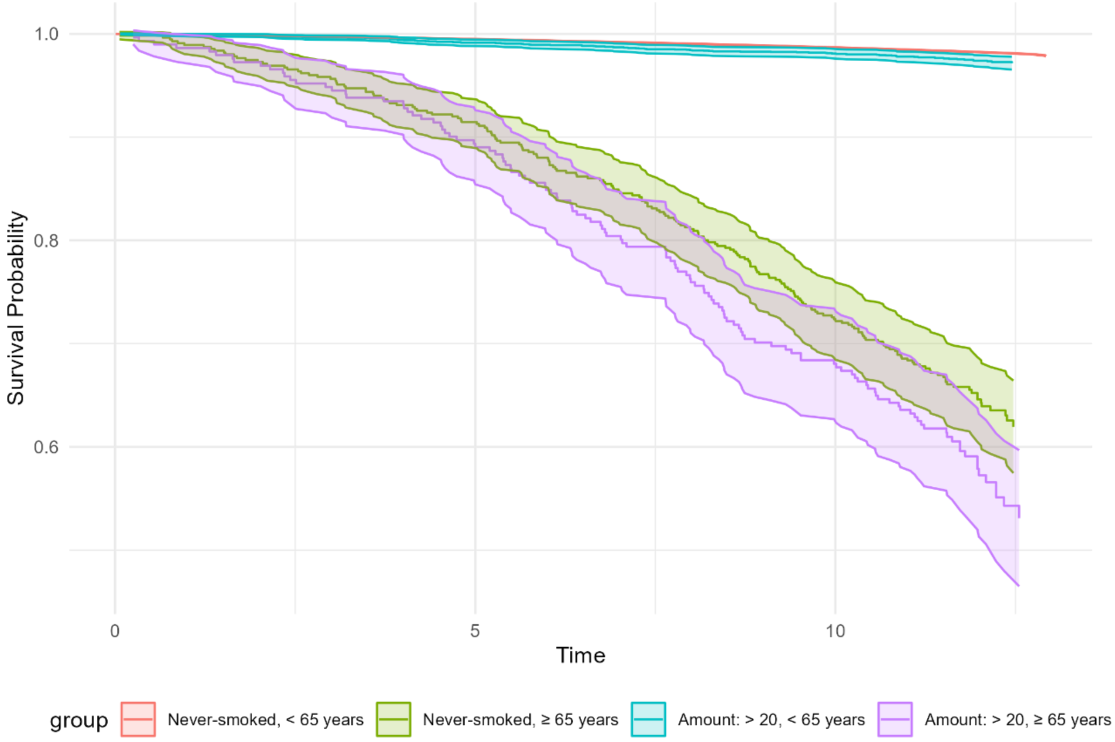

Figure S5.2 Kaplan-Meier curve by age and smoking intensity among women

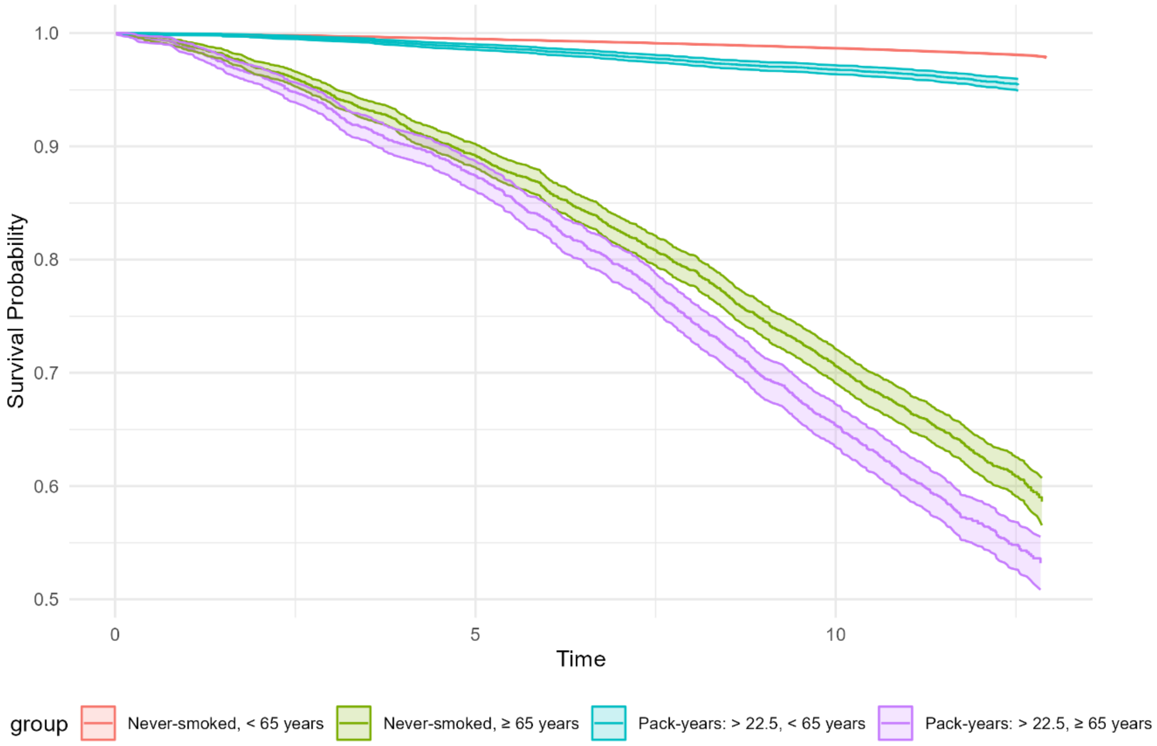

Figure S6.1 Kaplan-Meier curve by age and pack-year among men

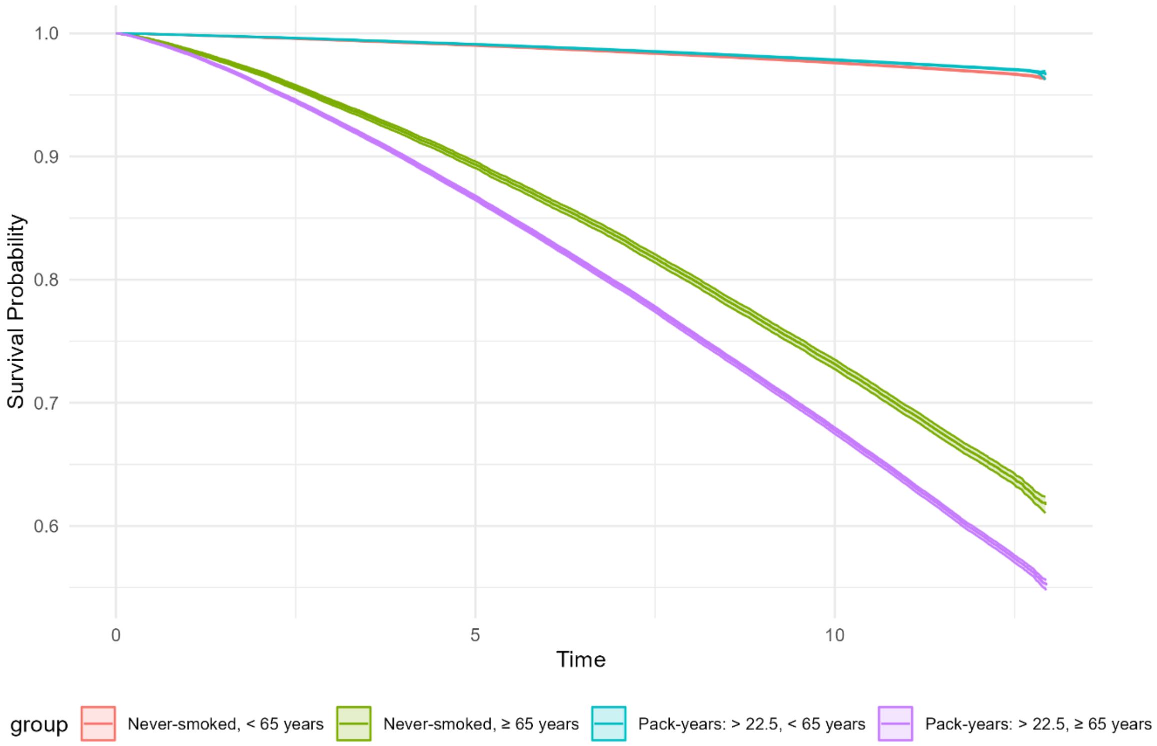

Figure S6.2 Kaplan-Meier curve by age and pack-year among women

Table S3 Hazard ratios, 95% confidence intervals, and the goodness-of-fits of models assessing the impacts of different smoking measures on all-cause deaths among all participants

**Smoking index**	**Men**					**Women**				
	**aHR (95% CI)***	**p-value**	**AIC**	**BIC**	**c-index**	**aHR (95% CI)***	**p-value**	**AIC**	**BIC**	**c-index**
Ever-smoking	1.07 (1.06, 1.08)	<0.0001	6467020	6467147	0.837	1.12 (1.09, 1.14)	<0.0001	3069422	3069541	0.853
Smoking status										
Never	Reference		6466446	6466583	0.840	Reference		3069415	3069543	0.853
Former	1.01 (1.00, 1.02)	0.0082				1.05 (1.00, 1.10)	0.0690			
Current	1.14 (1.13, 1.15)	<0.0001				1.14 (1.11, 1.18)	<0.0001			
Current smoking	1.13 (1.12, 1.14)	<0.0001	6466451	6466577	0.840	1.14 (1.11, 1.18)	<0.0001	3069416	3069535	0.853
Smoking duration (years)										
0 (never smoker)	Reference		6466361	6466520	0.837	Reference		3069421	3069569	0.853
≤ 10	0.95 (0.93, 0.96)	<0.0001				1.12 (1.07, 1.18)	<0.0001			
(10, 20]	1.00 (0.99, 1.01)	0.9453				1.19 (1.12, 1.26)	<0.0001			
(20, 25]	1.08 (1.06, 1.10)	<0.0001				1.17 (0.99, 1.39)	0.0681			
> 25	1.12 (1.11, 1.13)	<0.0001				1.09 (1.05, 1.12)	<0.0001			
Smoking intensity (cigarettes/day)										
0 (never smoker)	Reference		6466633	6466792	0.838	Reference		**3069413**	3069561	0.853
≤ 10	1.02 (1.01, 1.03)	0.0001				1.10 (1.07, 1.13)	<0.0001			
(10, 15]	1.04 (1.02, 1.06)	<0.0001				1.13 (1.00, 1.26)	0.0471			
(15, 20]	1.11 (1.10, 1.12)	<0.0001				1.16 (1.09, 1.23)	<0.0001			
> 20	1.16 (1.14, 1.18)	<0.0001				1.49 (1.28, 1.74)	<0.0001			
Pack-years										
0 (never smoker)	Reference		**6466335**	6466494	0.838	Reference		3069426	3069574	0.853
≤ 7.5	0.95 (0.94, 0.97)	<0.0001				1.1 (1.06, 1.15)	<0.0001			
(7.5, 15]	1.02 (1.01, 1.03)	0.0009				1.1 (1.05, 1.16)	<0.0001			
(15, 22.5]	1.08 (1.06, 1.09)	<0.0001				1.12 (1.04, 1.2)	0.0026			
> 22.5	1.13 (1.12, 1.14)	<0.0001				1.16 (1.1, 1.23)	<0.0001			

*All models adjusted for age, type of health insurance, income quintile, residential region, weekly alcohol consumption, weekly MVPA, and the body mass index.

Statistical significance at $p < 0.0017$

The lowest AIC value among all models for each sex is presented in **bold.**

Table S4 Effect size comparisons for models addressing all participants

Variable1	Variable2	HR1	SE1	HR2	SE2	ln(HR1)	ln(HR2)	ln(HR2- HR1)	SE diff *	Ratio of relative risks	95% CI		p value **
**Men**													
Ever smoking	Current smoking	1.07	0.004	1.13	0.004	0.07	0.12	0.05	0.006	1.06	1.04	1.07	*<0.0001*
Ever smoking	Smoking status	1.07	0.004	1.14	0.005	0.07	0.13	0.06	0.006	1.06	1.05	1.07	*<0.0001*
Ever smoking	Smoking duration	1.07	0.004	1.12	0.004	0.07	0.11	0.04	0.006	1.05	1.03	1.06	*<0.0001*
Ever smoking	Smoking intensity	1.07	0.004	1.16	0.008	0.07	0.15	0.08	0.009	1.08	1.06	1.10	*<0.0001*
Ever smoking	Pack-years	1.07	0.004	1.13	0.005	0.07	0.12	0.05	0.006	1.06	1.04	1.07	*<0.0001*
Current smoking	Smoking status	1.13	0.004	1.14	0.005	0.12	0.13	0.01	0.006	1.01	0.99	1.02	0.3666
Current smoking	Smoking duration	1.13	0.004	1.12	0.004	0.12	0.11	-0.01	0.006	0.99	0.98	1.00	0.1155
Current smoking	Smoking intensity	1.13	0.004	1.16	0.008	0.12	0.15	0.03	0.009	1.03	1.01	1.04	*0.0041*
Current smoking	Pack-years	1.13	0.004	1.13	0.005	0.12	0.12	0.00	0.006	1.00	0.99	1.01	0.8762
Smoking status	Smoking duration	1.14	0.005	1.12	0.004	0.13	0.11	-0.02	0.006	0.98	0.97	1.00	*0.0181*
Smoking status	Smoking intensity	1.14	0.005	1.16	0.008	0.13	0.15	0.02	0.009	1.02	1.00	1.04	*0.0294*
Smoking status	Pack-years	1.14	0.005	1.13	0.005	0.13	0.12	0.00	0.007	1.00	0.98	1.01	0.4790
Smoking duration	Smoking intensity	1.12	0.004	1.16	0.008	0.11	0.15	0.04	0.009	1.04	1.02	1.05	*0.0001*
Smoking duration	Pack-years	1.12	0.004	1.13	0.005	0.11	0.12	0.01	0.006	1.01	1.00	1.02	0.1015
Smoking intensity	Pack-years	1.16	0.008	1.13	0.005	0.15	0.12	-0.02	0.009	0.98	0.96	0.99	*0.0072*

Variable1	Variable2	HR1	SE1	HR2	SE2	ln(HR1)	ln(HR2)	ln(HR2- HR1)	SE diff *	Ratio of relative risks	95% CI		p value **
**Women**													
Ever smoking	Current smoking	1.12	0.013	1.14	0.015	0.11	0.13	0.02	0.020	1.02	0.98	1.06	0.2487
Ever smoking	Smoking status	1.12	0.013	1.14	0.015	0.11	0.13	0.02	0.020	1.02	0.98	1.07	0.2335
Ever smoking	Smoking duration	1.12	0.013	1.09	0.018	0.11	0.08	-0.03	0.022	0.97	0.93	1.02	0.2186
Ever smoking	Smoking intensity	1.12	0.013	1.49	0.078	0.11	0.40	0.29	0.079	1.34	1.14	1.56	*0.0003*
Ever smoking	Pack-years	1.12	0.013	1.16	0.029	0.11	0.15	0.04	0.032	1.04	0.98	1.11	0.2036
Current smoking	Smoking status	1.14	0.015	1.14	0.015	0.13	0.13	0.00	0.021	1.00	0.96	1.04	0.9715
Current smoking	Smoking duration	1.14	0.015	1.09	0.018	0.13	0.08	-0.05	0.023	0.95	0.91	1.00	*0.0312*
Current smoking	Smoking intensity	1.14	0.015	1.49	0.078	0.13	0.40	0.27	0.080	1.30	1.12	1.53	*0.0008*
Current smoking	Pack-years	1.14	0.015	1.16	0.029	0.13	0.15	0.02	0.033	1.02	0.95	1.08	0.5965
Smoking status	Smoking duration	1.14	0.015	1.09	0.018	0.13	0.08	-0.05	0.023	0.95	0.91	0.99	0.0287
Smoking status	Smoking intensity	1.14	0.015	1.49	0.078	0.13	0.40	0.27	0.080	1.30	1.12	1.52	*0.0009*
Smoking status	Pack-years	1.14	0.015	1.16	0.029	0.13	0.15	0.02	0.033	1.02	0.95	1.08	0.6129
Smoking duration	Smoking intensity	1.09	0.018	1.49	0.078	0.08	0.40	0.32	0.080	1.37	1.17	1.61	*0.0001*
Smoking duration	Pack-years	1.09	0.018	1.16	0.029	0.08	0.15	0.07	0.034	1.07	1.00	1.14	*0.0466*
Smoking intensity	Pack-years	1.49	0.078	1.16	0.029	0.40	0.15	-0.25	0.083	0.78	0.66	0.92	*0.0028*

*sqrt(SE1^2 + SE2^2)
**Statistically significant p-values ($p<0.05$) are *italicized*

REFERENCES

1. Leffondré K, Abrahamowicz M, Siemiatycki J, Rachet B. Modeling smoking history: a comparison of different approaches. *Am J Epidemiol*. Nov 1 2002;156(9):813-23. doi:10.1093/aje/kwf122

2. Nance R, Delaney J, McEvoy JW, et al. Smoking intensity (pack/day) is a better measure than pack-years or smoking status for modeling cardiovascular disease outcomes. *Journal of clinical epidemiology*. 2017;81:111-119. doi:10.1016/j.jclinepi.2016.09.010

3. Bhatt SP, Kim Y-i, Harrington KF, et al. Smoking duration alone provides stronger risk estimates of chronic obstructive pulmonary disease than pack-years. *Thorax*. 2018;73(5):414-421. doi:10.1136/thoraxjnl-2017-210722

4. Hudson M, Lo E, Baron M, Steele R, Canadian Scleroderma Research G. Modeling smoking in systemic sclerosis: A comparison of different statistical approaches. https://doi.org/10.1002/acr.20416. *Arthritis Care & Research*. 2011/04/01 2011;63(4):570-578. doi:https://doi.org/10.1002/acr.20416

5. Etter J-F, Perneger TV. Measurement of self reported active exposure to cigarette smoke. *J Epidemiol Community Health*. 2001;55(9):674-680. doi:10.1136/jech.55.9.674

6. Remen T, Pintos J, Abrahamowicz M, Siemiatycki J. Risk of lung cancer in relation to various metrics of smoking history: a case-control study in Montreal. *BMC Cancer*. Dec 19 2018;18(1):1275. doi:10.1186/s12885-018-5144-5

7. Lubin JH, Albanes D, Hoppin JA, et al. Greater coronary heart disease risk with lower intensity and longer duration smoking compared with higher intensity and shorter duration smoking: congruent results across diverse cohorts. *Nicotine & Tobacco Research*. 2017;19(7):817-825. doi:10.1093/ntr/ntw290

8. Lubin JH, Caporaso NE. Misunderstandings in the misconception on the use of pack-years in analysis of smoking. Letter. *Br J Cancer*. Mar 2013;108(5):1218-1220. doi:10.1038/bjc.2013.76

9. Thomas DC. Invited Commentary: Is It Time to Retire the "Pack-Years" Variable? Maybe Not! Editorial Material. *Am J Epidemiol*. Feb 2014;179(3):299-302. doi:10.1093/aje/kwt274

10. Leffondré K, Abrahamowicz M, Xiao Y, Siemiatycki J. Modelling smoking history using a comprehensive smoking index: application to lung cancer. *Stat Med*. Dec 30 2006;25(24):4132-46. doi:10.1002/sim.2680

11. Leffondré K, Abrahamowicz M, Xiao Y, Siemiatycki J. Modelling smoking history

using a comprehensive smoking index: application to lung cancer. https://doi.org/10.1002/sim.2680. *Stat Med*. 2006/12/30 2006;25(24):4132-4146. doi:https://doi.org/10.1002/sim.2680

12. Ogawa K, Kawaguchi T, Koh Y, et al. Can smoking duration alone replace pack-years to predict the risk of smoking-related oncogenic mutations in non-small cell lung cancer (NSCLC)? 2019;doi:10.1136/bmjopen-2019-035615

13. Pleasants RA, Rivera MP, Tilley SL, Bhatt SP. Both duration and pack-years of tobacco smoking should be used for clinical practice and research. *Annals of the American Thoracic Society*. 2020;17(7):804-806. doi:10.1513/AnnalsATS.202002-133VP

14. Flanders WD, Lally CA, Zhu B-P, Henley SJ, Thun MJ. Lung Cancer Mortality in Relation to Age, Duration of Smoking, and Daily Cigarette Consumption: Results from Cancer Prevention Study II1. *Cancer Res*. 2003;63(19):6556-6562.

15. Lubin JH, Caporaso NE. Cigarette smoking and lung cancer: modeling total exposure and intensity. *Cancer Epidemiol Biomarkers Prev*. Mar 2006;15(3):517-23. doi:10.1158/1055-9965.Epi-05-0863

16. Lubin JH, Couper D, Lutsey PL, Woodward M, Yatsuya H, Huxley RR. Risk of Cardiovascular Disease from Cumulative Cigarette Use and the Impact of Smoking Intensity. *Epidemiology*. 2016;27(3)

17. Young JC, Paul NJ, Karatas TB, et al. Cigarette smoking intensity informs outcomes after open revascularization for peripheral artery disease. *J Vasc Surg*. 2019/12/01/ 2019;70(6):1973-1983.e5. doi:https://doi.org/10.1016/j.jvs.2019.02.066

18. Hoffmann K, Bergmann MM. RE: "MODELING SMOKING HISTORY: A COMPARISON OF DIFFERENT APPROACHES". *Am J Epidemiol*. 2003;158(4):393-393. doi:10.1093/aje/kwg159

19. Hoffmann K, Krause C, Seifert B. The German Environmental Survey 1990/92 (GerES II): primary predictors of blood cadmium levels in adults. *Arch Environ Health*. Jul-Aug 2001;56(4):374-9. doi:10.1080/00039890109604471

© 2025 Kang H. et al.
